# Long‐term shifts in the functional diversity of abandoned wet meadows: Impacts of historical disturbance and successional pathways

**DOI:** 10.1002/ece3.8186

**Published:** 2021-10-12

**Authors:** Patryk Czortek, Lidia Borkowska, Marlena Lembicz

**Affiliations:** ^1^ Białowieża Geobotanical Station Faculty of Biology University of Warsaw Białowieża Poland; ^2^ Faculty of Natural Sciences Siedlce University of Natural Sciences and Humanities Siedlce Poland; ^3^ Faculty of Biology Department of Plant Taxonomy Adam Mickiewicz University in Poznań Poznań Poland

**Keywords:** functional composition change, historical disturbance, interspecific competition, long‐term vegetation resurvey, successional scenario

## Abstract

Investigating the direction of changes in functional diversity involving successional pathways and historical disturbances may be a promising tool for predictions of the effectiveness of the seminatural meadows conservation, with great emphasis on formulation of more cost‐effective restoration strategies. The goal of this research was to assess the differences in long‐term shifts in the functional diversity of plant species in seminatural wet meadows unmanaged for the last 40 years, under the influence of different successional pathways and historical disturbances. Using ordination techniques, linear mixed‐effect models, a set of plant functional traits and parameters of functional diversity, we assessed the importance of habitat filtering, competition, and niche partitioning in shaping community assembly changes over time. The most dramatic shifts in functional diversity were found in the *Carex acutiformis* successional pathway after topsoil removal, where colonization by successional inhibitors was the main driver causing decreases in functional dispersion and divergence. This was expressed as a decrease in the importance of habitat filtering and replacement of specialized species by competitors with heavier seeds and higher specific leaf area. Regarding the *C*. *cespitosa* and *Salix cinerea* pathways, the magnitudes of shifts in functional diversity were milder and differed less between the historical topsoil removal and mowing treatments, thereby maintaining a large role for niche partitioning in shaping the vegetation structure. The results of our study highlight the importance of tussock sedges and shrubs as effective buffers against the functional homogenization of meadows driven by the decreases in functional diversity of plant species, even from a long‐term perspective.

## Graphical abstract

The goal of this research was to assess the differences in long‐term shifts in the functional diversity of plant species in seminatural wet meadows unmanaged for the last 40 years, under the influence of different successional pathways and historical disturbances. The most dramatic shifts in functional diversity were found in the *Carex acutiformis* successional pathway after topsoil removal, where colonization by successional inhibitors was the main driver causing decreases in functional dispersion and divergence. Regarding the *C*. *cespitosa* and *Salix cinerea* pathways, the magnitudes of shifts in functional diversity were milder and differed less between the historical topsoil removal and mowing treatments, thereby maintaining a large role for niche partitioning in shaping the vegetation structure. The results of our study highlight the importance of tussock sedges and shrubs as effective buffers against the biotic homogenization of meadows driven by the simplification of functional composition, even from a long‐term perspective.

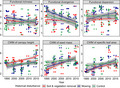

## 1 INTRODUCTION

From the entire spectrum of global grassland biomes, European seminatural meadows are considered one of the most important plant species diversity hot spots (Dengler et al., 2014). Historically, the maintenance of outstandingly high taxonomic and functional diversities of seminatural meadows was possible due to a long tradition of extensive use (Habel et al., 2013). However, since the middle of the 20th century, areas covered by seminatural meadows have declined (Török & Dengler, 2018), making these ecosystems one of the most threatened worldwide (Edwards & Kučera, 2019). In Europe, during the last century, the area occupied by seminatural ecosystems was reduced to about 97% of their historical range (Sammul et al., 2008). One negative effect of socioeconomic transformation of meadows is the replacement of specialized plant species by generalists (Cardinale et al., 2012). An outcome of this process is biotic homogenization of plant communities (Price et al., 2020), leading to simplification of both taxonomical diversity and functional diversity of vegetation (Olden et al., 2018). This may be expressed in increasing compositional similarity among historically divergent plant communities (Cholewińska et al., 2020; Reinecke et al., 2014; Sonnier et al., 2014), which in further perspective can alter the trophic net structure (Dainese et al., 2016) and reduce the capacity of ecosystems for provisioning of the ecosystem services (Edwards & Kučera, 2019). While taxonomical homogenization of plant communities was addressed by numerous earlier studies (e.g., Cholewińska et al., 2020; Price et al., 2020), mechanisms of functional homogenization (i.e., losses of functional diversity over time) are still poorly understood (e.g., Olden et al., 2018; Reinecke et al., 2014; Sonnier et al., 2014).

The increasing number of reports on the temporal shifts in species composition and on effective methods of conservation of seminatural meadows in relation to different gradients of disturbances and land use (e.g., mowing or vegetation removal) demonstrate the importance of this topic for ecological research over the past decades (e.g., Doležal et al., 2018; Tonn et al., 2019). Apart from numerous previous studies, which examined long‐term shifts in taxonomic composition (e.g., Mangano et al., 2019), a stronger emphasis has been placed recently on shifts in the functional trait community composition of abandoned seminatural meadows (e.g., Doležal et al., 2019; Kahmen & Poschlod, 2004; Mudrák et al., 2019; Velbert et al., 2017). Assessments of the functional diversity of plant communities provide the basis for understanding shifts in species composition, dynamics of biodiversity, and ecosystem functioning (Díaz et al., 2007). In this light, the examination of functional diversity enables the identification of the main ecological mechanisms (i.e., environmental filtering, competition, and niche partitioning) that shape the community assembly rules (Czortek et al., 2021). The functional diversity approach was found to be useful in the assessments of the relationships between the structure of seminatural meadow ecosystems and management practices (Engst et al., 2016). Except for reports that examined seasonal dynamics of functional diversity under the implementation of different mowing regimes (e.g., Doležal et al., 2019), other authors studied the mechanisms of succession on unmanaged, seminatural meadows (e.g., Conradi & Kollmann, 2016; Kahmen & Poschlod, 2004; Rosenthal, 2010). It is well known that the cessation of land use may lead to a decline in the overall functional diversity of meadows during succession, but ranges of these shifts depend on historical management type (e.g., Niu et al., 2016; Tardella & Catorci, 2015). On the other hand, the implementation of moderate disturbance (e.g., rotavation‐driven, vegetation removal) can increase functional diversity and enable the coexistence of pioneer and specialized plant species that follow different life strategies (e.g., Schnoor et al., 2015). In contrast, selective removal of dominant species seems to exert a relatively low impact on the temporal and seasonal dynamics of plant functional composition in European wet meadows of the temperate zone of climate (e.g., Doležal et al., 2019). Other authors, in turn, found a combination of mowing and moderate topsoil removal to be the most efficient land‐use type to maintain high meadow diversity (Resch et al., 2019). Velbert et al. (2017), for instance, reported the largest losses in overall functional diversity in the early stages of succession, which were explained by the increasing niche separation of foliar traits connected with competitive abilities, while implementation of mowing drove a divergence in reproductive traits. Other authors (e.g., Kahmen & Poschlod, 2004) demonstrated that in subsequent years of succession, nongrazed meadows have been dominated by plants with higher specific leaf area (SLA) and seed mass, thus revealing an increasing importance of biotic factors, but a decreasing role of habitat filters in shaping the community structure.

To our knowledge, there is a large lack of long‐term surveys focusing on shifts in the functional diversity of abandoned wet meadows, while simultaneously comparing the pace and course of different successional pathways under different types of historical disturbances (compare, e.g., Kahmen & Poschlod, 2004; Niu et al., 2016; Schnoor et al., 2015). Implementation of this approach in the conservation of wet meadows may be especially important for predicting the roles of both abiotic and biotic factors in influencing shifts in community assembly (Busch et al., 2019). Therefore, it may provide more mechanistic insights into the responses of the individual target species based on their life‐history traits and realized niches (Díaz et al., 2007). Furthermore, it can be especially relevant in the determination of the performance of the entire guilds of species. This, in turn, may allow experimentally based assessments of conservation priorities and, therefore, formulation of more cost‐effective strategies in the restoration of wet meadows, which are regarded as very rare communities in Europe (Diekmann et al., 2019). Furthermore, in a vast number of cases the perspectives of their recovery seem to be insufficient due to often small extinction debt revealed by many meadow target species (Otsus et al., 2014; Waldén et al., 2017).

A classical study area characterized by a long tradition of land use, and a long‐term sequence of detailed investigations on successional dynamics, is seminatural wet meadows of the Reski Range (NE Poland). A previous series of long‐term permanent plot surveys, which were initiated in 1972 by K. Falińska, determined three main successional pathways prevailing in this area (Falińska, 2003). This created an opportunity to establish in 1995 a new, long‐term “inverse” experiment aiming to examine how the mechanisms of species turnover differ among successional pathways and types of historical disturbance. The main goals of the present study were to assess (i) long‐term shifts in the importance of habitat filtering, competition, and niche partitioning in shaping the functional composition of wet unmanaged meadows, (ii) whether the magnitude of these shifts differs between successional pathways and between different types of historical disturbances, and (iii) which pathway of succession and historical disturbance type would be most promising regarding the potential restoration of abandoned wet meadows.

## 2 MATERIALS AND METHODS

### 2.1 Study area

The study was conducted on unmown meadows in the Reski Range, with an area of approximately 15 ha. The Reski Range is located in the north western part of the Białowieża Glade in the Białowieża National Park (N 52°42′32″; E 23°50′06″) in the Narewka River valley (NE Poland; 150 m a.s.l.). The Reski Range is located in the zone of transitional temperate climate with domination of continental influences, with mean annual temperature of about 6.8°C and mean annual precipitation of 633 mm (Żarnowiecki, 2008). Meadows of the Reski Range occupy the habitats of former ash‐alder riparian forests, developing on mineral and organogenic soils (peaty or clayey silt‐laden soils; Malhzan et al., 2009). More than 200 years ago, in that part of the Narewka River valley, forests were cut down, and extensively mowed meadows were formed (Falińska, 2003). Due to relatively low utility values and early spring flooding, the meadows in the Reski Range were gradually abandoned by farmers; abandonment began in the 1960s and ended in 1978, followed by spontaneous secondary succession. Recently, nonforest vegetation of the Reski Range consists of tall grass communities (dominated by *Phragmites australis* or *Phalaris arundinacea*), tall herb communities with *Lysimachia vulgaris* and *Filipendula ulmaria*, and small patches of wet grasslands with *Cirsium rivulare* (Borkowska, 2016). This created an opportunity to establish a long‐term permanent plot experiments, aiming to assess the population dynamics of single plant species over time, as well as the mechanisms of the replacement of plants during secondary succession (Falińska, 1991). After approximately 20 years of these detailed investigations, K. Falińska identified three main pathways, based on life‐history traits (e.g., clonal architecture, seed bank characteristics, and reproductive strategies) of the dominant species (with more than 80% of coverage in plots at the beginning of the experiment) and co‐occurring plants (Falińska, 2003). Before the experiment establishment, three successional pathways studied differed not only in the dominant species but also in the composition of the species composition (Borkowska, 2004). The pathway of succession, with the participation of *Carex acutiformis* (hereafter “*C*. *acutiformis* pathway”), involves the dominance of species typical of rush communities. Before the beginning of the experiment, the dominant species there were nitro‐ and hygrophilous tall herbs, that is, *C*. *acutiformis*, *F*. *ulmaria*, and *L*. *vulgaris*, while *Carex cespitosa* and *Cirsium palustre* occurred there sporadically. *Salix cinerea* succession pathway (hereafter “*S*. *cinerea* pathway”) involves a changing species composition according to the following scheme: meadow–tall herb communities–thicket–forest. Before the beginning of the experiment, apart from dominance of *S*. *cinerea* in the shrub layer, a relatively high abundance of *S*. *pentandra* shrub was recorded, while *C*. *acutiformis* and *F*. *ulmaria* were less abundant. Remaining species, for example, *L*. *vulgaris*, *C*. *cespitosa*, *C*. *palustre*, *C*. *rivulare*, *Geum rivale*, *Viola* spp., and *Lotus corniculatus*, occurred within this scenario only sporadically. In turn, the *Carex cespitosa* (hereafter “*C*. *cespitosa* pathway”) pathway involves coexistence of species composition characteristics of rush, tall herb, and thicket communities, with the highest abundance (before the beginning of the experiment) recorded for: *C*. *cespitosa*, *C*. *appropinquata*, *F*. *ulmaria*, *C*. *palustre*, *G*. *rivale*, *Angelica sylvestris*, and *Equisetum* spp. (Borkowska, 2004; Falińska, 2003).

### 2.2 Experimental design

An experiment was conducted in 1995–2015, approximately 20 years after the initial long‐term research on plant demography during secondary succession (Falińska, 1991, 2003). To study each of the three successional pathways identified by K. Falińska as the most common in the study area, one transect consisting of 30 permanent plots (1 × 1 m) was established and divided into three subtransects, each one being 10 m in length and 1 m in width, with the distance 50 m between each subtransect (i.e., successional pathway). Therefore, the total number of permanent plots for the three successional pathways and three treatments was 90. At the beginning of the experiment, two different types of disturbances were applied once in each subtransect for each successional pathway. The first subtransect was subjected to topsoil removal disturbance (*n* = 10): The plants, including roots, and topsoil were removed to the depth of 25 cm. In the second subtransect (*n* = 10), mowing disturbance was applied: The aerial parts of the plants were cut down with a knife/scythe. The third subtransect (*n* = 10) was left intact as a control area, without the application of any treatment. Thus, as each successional pathway was identified prior to application of different treatments, we assumed that observations within each subtransect were independent. Permanent plots were established in 1994, and the first field survey took place in 1995. Then, within five‐year intervals (to 2015), analyses of species composition were carried out on each plot (1 × 1 m) within each subtransect and each successional pathway, and species abundance (the total number of ramets per species recorded) was noted. Plant species names were adopted after Mirek et al. (2002).

One should expect that the consideration of only one replicate per successional pathway is the main limitation of our study, making the results highly site‐specific and generating pseudoreplications, thus affecting low transferability of the patterns revealed to other wet meadows of similar character. However, at the start of the vegetation survey in 1995, this original and unique experimental layout was designed especially for the examination of long‐term shifts in plant demography and vegetation. Thus, issues connected with the quality of experimental design and quality of historical data appear very frequently in vegetation resurvey studies, where the initial data were collected to achieve completely different aims compared with the research questions posed by recent researchers (e.g., Czortek, Eycott, et al., 2018). However, long‐term studies conducted on permanent plots seem to be of greatest importance, as these data series constitute a unique source of information on historical vegetation. Therefore, these data can be used as a basis for future examination of shifts in species composition under the influence of anthropogenic drivers, such as climate warming or land‐use change (Stöckli et al., 2011). Moreover, the application of this approach, even with a low number of replications, makes it possible to obtain results that are not biased by random factors, that is, observer errors. In our case, the field study, from the beginning to the end of the experiment, was carried out by only one researcher, that is, highly experienced in plant ecology and plants identification (LB), which is rather a very rare phenomenon in resurvey studies (Verheyen et al., 2018). In addition, despite some limitations, this experiment made it possible to omit all uncertainties connected with plot relocation, as the majority of resampling studies are performed on quasi‐ or even nonpermanent plots (Czortek, Dyderski, et al., 2020; Kapfer et al., 2017).

### 2.3 Functional traits and functional diversity indices

Using the LEDA database, we compiled information on seven functional traits related to plant size and leaf morphology (i.e., canopy height [m], shoot growth form [eight categories], leaf distribution along stem [five categories], and specific leaf area; SLA [mm^2^/mg]) and dispersal abilities (dispersal mode [46 categories], seed mass [mg], and seed bank longevity [two categories]). Traits related to plant size and morphology describe the competitive abilities of plant species and provide information on strategies for the acquisition, usage, and distribution of resources among different organs. Traits related to dispersal abilities are reflections of reproductive strategies, seed dispersion distances, survival, and responses of plant species to disturbances.

One should expect that usage of plant functional traits provided by trait databases may not allow comparison of shifts in functional diversity over time between treatments and successional pathways in a small spatial scale, and under specific combination of microhabitat conditions. Thus, the same plant species growing under different treatments may differ substantially in terms of particular functional trait values, resulting in high intraspecific trait variability. From these reasons, employment of trait databases in ecological research is considered as promising approach in studies conducted rather in larger spatial scales (e.g., Maes et al., 2019; Thonicke et al., 2020; Vanneste et al., 2019). Moreover, according to Paź‐Dyderska et al. (2020), intraspecific trait variability of selected functional traits of 167 plant species provided by, for instance, the LEDA database (e.g., SLA, total leaf area, and leaf mass), is underestimated when compared to direct field measurements. However, the same authors explored that interspecific variability of functional traits (triggered by species identity) is bigger than intraspecific variability (an outcome of site‐specific factors). In addition, in their study, site‐specific random factors explained lower amounts of variation in traits than species identity. Thus, this may support the usage of plant functional traits obtained from trait databases as a proper tool in survey of temporal shifts in plant species functional diversity, even in small spatial scales. On the other hand, numerous previous studies conducted in small spatial scales found the usage of data base‐derived traits highly informative in assessments of the role of different ecological mechanisms in shaping plants coexistence patterns (e.g., Czortek, Czortek, et al., 2020b; Czortek & Pielech, 2020; Dyderski et al., 2016; Jagodziński et al., 2017) or changes in their importance over time (e.g., Czortek et al., 2018; Czortek, Dyderski, et al., 2020). However, as we did not measure intraspecific trait variability, which in our case can be presumably high due to high dependence of plant species composition on site‐specific factors, our results should be interpreted with a caution. To explore temporal changes in the importance of habitat filtering, as well as competition and niche partitioning in shaping the community assembly, for each experimental plot (in each of the five‐time periods of the vegetation resurvey) across each type of historical disturbance and successional pathway, we calculated (i) community‐weighted means (CWMs) of functional traits, that is, canopy height, seed mass, and SLA; and (ii) three indices of functional diversity, that is, functional richness, functional divergence, and functional dispersion. Functional richness and functional divergence were calculated following Villéger et al. (2008), while functional dispersion was calculated following Laliberté and Legendre (2010). These indices characterize the distribution of plant species' life‐history traits within the plant community hyperspace (Laliberté & Legendre, 2010). Functional richness measures the filled niche size, and low values of this parameter are an expression of a low degree of occupancy of the niche space by plants with different traits (Villéger et al., 2008). Therefore, low values of functional richness may indicate that habitat filtering or competition is the main mechanisms for shaping the plant community structure. Both functional divergence and functional dispersion provide information on distances between functional traits carried by different species in the plant community trait hyperspace (Mason et al., 2005; Villéger et al., 2008). Functional divergence measures the average distance of each trait to the center of gravity (center space) of the trait space, and functional dispersion measures the same distance to the centroid (center point) of all traits (Hedberg et al., 2014). In this context, functional divergence is more sensitive to species with extreme values of different functional traits, providing information on the importance of specialized species with narrow realized niches in shaping the community structure (Kotowski et al., 2013). High values of functional divergence and functional dispersion express vast distances between functional traits carried by different species, suggesting that the richness of functional traits in the plant community trait hyperspace is high, which, in turn, provides information on the high importance of niche partitioning in shaping community assembly (Carroll et al., 2011).

### 2.4 Data analysis

We conducted all analyses using R software (R Core Team, 2019). To explore the main shifts in the species composition of meadow vegetation over time and between three treatments (topsoil removal, mowing, and control) for each of the three successional pathways, we performed one nonmetric multidimensional scaling ordination (NMDS) with pairwise Bray–Curtis dissimilarity matrices. Using a passive projection of vectors for each of the three successional pathways, we assessed the indirect relationships between species composition of the experimental plots and indices of functional diversity and CWMs of plant functional traits. Using a permutation test with 999 iterations for each vector, we calculated the determination coefficient *R* ^2^ and *p*‐values.

To assess direct shifts in values of functional diversity indices and CWMs of plant functional traits over time and between historical disturbance types, we used linear mixed‐effect models (LMMs). Prior to LMMs, we checked whether the response variables represent close‐to‐normal distributions. For each vegetation parameter with respect to each successional pathway, we performed one linear regression model (without random factors) and one diagnostic plot (*base*::*plot(lm())* function) visualizing relationship between theoretical quantiles and standardized residuals (Appendix S1). Visual inspection of diagnostic plots revealed rather weak deviations from ideal relationship (dashed gray lines; Appendix S1) in case of majority of response variables analyzed. This provided an evidence that majority of response variables represent rather close‐to‐normal distributions. Despite some significant deviations reported (e.g., for: CWM of seed mass, or CMW of specific leaf area in “*S*. *cinerea* scenario”), we assumed the close‐to‐normal distribution for all analyzed parameters. We made this assumption in order to make the results comparable between different treatments and succession scenarios, as well as to minimize uncertainties in interpretation of the results, connected with the employment of different regression methods. From these reasons, in our study we emphasized more the ecological significance of the results, by using the most parsimonious and joint methods, rather than more sophisticated procedures (e.g., generalized additive models; Nakagawa & Cuthill, 2007; Wasserstein & Lazar, 2016). Thus for each vegetation parameter with respect to each successional pathway, one LMM was performed. We decided to employ LMMs to account for random effects linked with plot‐specific effects, that is, due to the lack of independent replications within a single pathway of succession, as well as to address potential effects of pseudoreplications (which could be produced as an outcome of our experimental design) on the results obtained. To assess the fixed effects related to the year of observation and historical disturbance type, we calculated marginal ($R_{m}^{2}$) and conditional ($R_{c}^{2}$) coefficients of determination. $R_{m}^{2}$ indicates how much of the variance is explained by fixed effects only, and $R_{c}^{2}$ indicates the amount of variance explained by both fixed and random effects. Therefore, the difference between conditional and marginal coefficients of determination provides information on the amount of variance explained by the random effects only. To account for nonlinear relationships related to the uneven ranges among response variables, data were scaled (divided by the standard deviation) prior to the analyses. The ecological significance of the results was revealed by focusing more on the effect sizes and directions of relationships than on the *p*‐values and *R* ^2^ determination coefficients (Steel et al., 2013).

## 3 RESULTS

**FIGURE 1:**
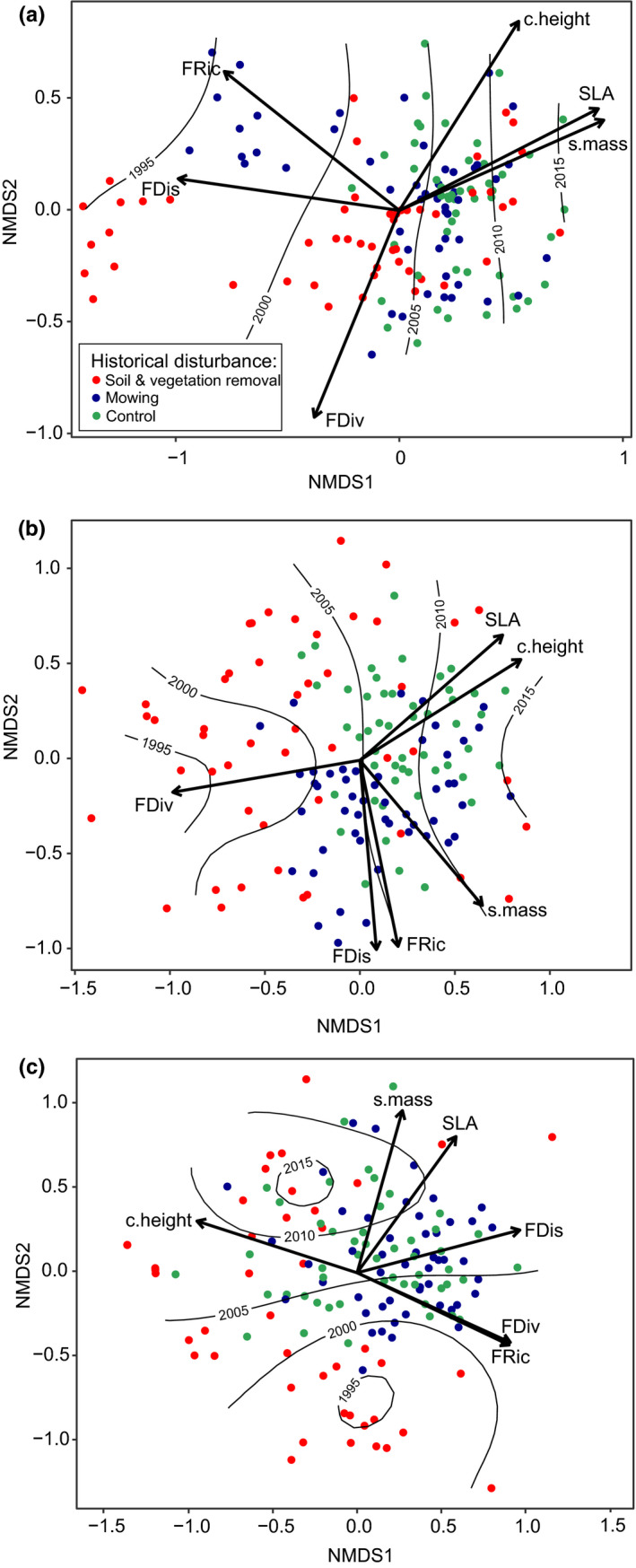
Results of NMDS ordinations showing changes in the functional composition of meadow vegetation over time (black isolines) and between different types of historical disturbances regarding the “*Carex acutiformis*” (a), “*Salix cinerea*” (b), and “*Carex cespitosa*” successional scenarios (c) with passive projection (black arrows) of the functional diversity parameters (Table 1): FRic—functional richness, FDiv—functional divergence, FDis—functional dispersion and CWMs (community‐weighted means) of plant functional traits: c.height—CWM of canopy height, s. mass—CWM of seed mass, SLA—CWM of specific leaf area. Points represent plots

We found significant changes in the species composition of meadow vegetation over the years from 1995 to 2015 (Figure 1; Table 1). The highest compositional dissimilarities over time and between historical disturbance types we found in the “*C*. *acutiformis* pathway” (Figure 1a). Regarding the “*S*. *cinerea*” and “*C*. *cespitosa*” pathways, the magnitudes of temporal shifts in species composition, as well as compositional dissimilarities between historical treatments, were lower than those observed for the “*C*. *acutiformis* pathway” (Figure 1b,c). Considering plots with topsoil removal and mowing in the “*C*. *acutiformis* pathway,” the values of functional dispersion and functional richness decreased significantly in the subsequent years of the experimental survey. At the same time, the proportions of species with higher canopy height, seed mass, and SLA increased significantly over time under the conditions of both historical treatments (Figure 1a). In the “*S*. *cinerea* pathway” and under conditions of topsoil removal disturbance, only the functional divergence decreased significantly in subsequent years of the experiment. In this pathway, taking into account both topsoil removal and mowing disturbances, we found significant increases in the proportions of species of higher SLA and canopy heights over time (Figure 1b). Regarding the “*C*. *cespitosa* pathway,” the contribution of plant species with higher SLA and producing heavier seeds increased significantly over the experimental survey period, but this increase was mostly related to plots representing the topsoil‐disturbance type (Figure 1c).

**TABLE 1:** Significance of vegetation parameters describing temporal shifts in species composition and functional diversity of plants among three successional scenarios

Parameter	Abbreviation	NMDS1	NMDS2	*R* ^2^	*p*
*“Carex acutiformis”* succession scenario					
Functional richness	FRic	−0.783	0.621	0.487	.**001**
Functional divergence	FDiv	−0.380	−0.924	0.306	.**001**
Functional dispersion	FDis	−0.989	0.141	0.391	.**001**
CWM of canopy height	c.height	0.533	0.845	0.168	.**001**
CWM of seed mass	s.mass	0.914	0.403	0.663	.**001**
CWM of specific leaf area	SLA	0.890	0.455	0.573	.**001**
Year	—	0.991	−0.130	0.457	.**001**
Historical disturbance	—	—	—	0.207	.**001**
*“Salix cinerea”* succession scenario					
Functional richness	FRic	0.201	−0.979	0.418	.**001**
Functional divergence	FDiv	−0.985	−0.168	0.237	.**001**
Functional dispersion	FDis	0.087	−0.996	0.230	.**001**
CWM of canopy height	c.height	0.847	0.530	0.144	.**001**
CWM of seed mass	s.mass	0.645	−0.764	0.029	.122
CWM of specific leaf area	SLA	0.751	0.659	0.497	.**001**
Year	—	0.997	0.073	0.404	.**001**
Historical disturbance	—	—	—	0.226	.**001**
*“Carex cespitosa”* succession scenario					
Functional richness	FRic	0.903	−0.428	0.143	.**001**
Functional divergence	FDiv	0.910	−0.413	0.087	.**005**
Functional dispersion	FDis	0.967	0.253	0.359	.**001**
CWM of canopy height	c.height	−0.951	0.307	0.479	.**001**
CWM of seed mass	s.mass	0.269	0.963	0.145	.**001**
CWM of specific leaf area	SLA	0.587	0.809	0.147	.**001**
Year	—	−0.195	0.980	0.381	.**001**
Historical disturbance	—	—	—	0.106	.**001**

Note

Apart from time (presented in Figure 1 as black isolines) and historical disturbance (presented in Figure 1 as different colors), remaining parameters were passively fitted as vectors (arrows) to the results of the NMDS ordinations (Figure 1). NMDS1 and NMDS2 are coordinates of parameters along first and second ordination axes. Determination coefficients *R* ^2^ and *p*‐values were estimated using permutation tests with 999 iterations. Significant results are in bold.

**FIGURE 2:**
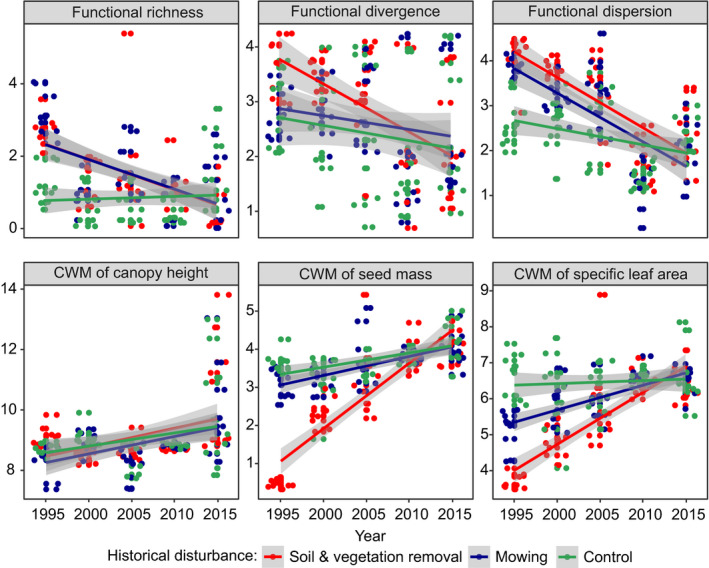
Visualization of linear mixed‐effect models showing changes in the functional composition of meadow vegetation over time and between different types of historical disturbances in the “*Carex acutiformis*” successional pathway. Gray backgrounds are standard errors. Note that data were scaled prior to the analyses. Points represent plots. For model parameters, see Table 2

**FIGURE 3:**
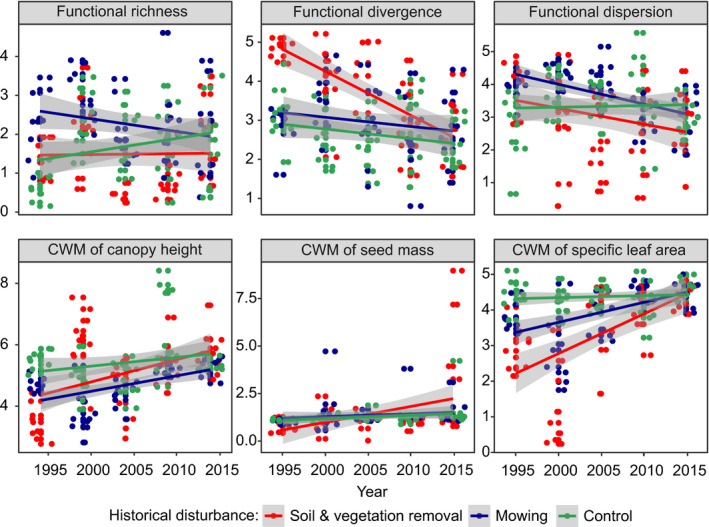
Visualization of linear mixed‐effect models showing changes in the functional composition of meadow vegetation over time and between different types of historical disturbances in the “*Salix cinerea*” successional pathway. Gray backgrounds are standard errors. Note that data were scaled prior to the analyses. Points represent plots. For model parameters, see Table 3

**FIGURE 4:**
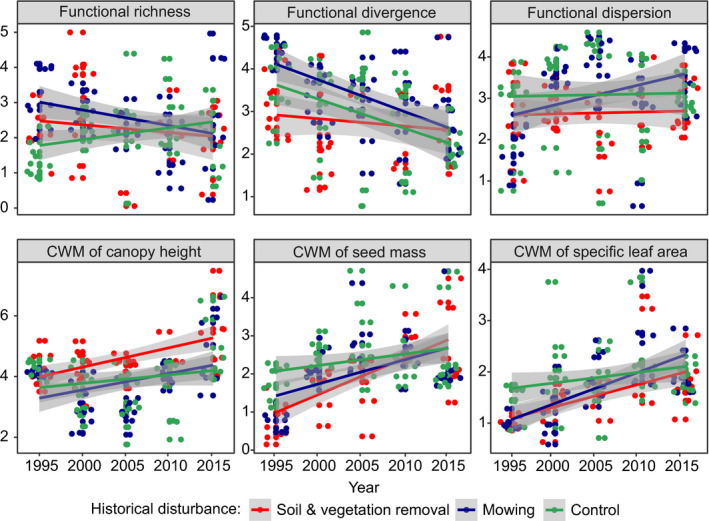
Visualization of linear mixed‐effect models showing changes in the functional composition of meadow vegetation over time and between different types of historical disturbances in the “*Carex cespitosa*” successional pathway. Gray backgrounds are standard errors. Note that data were scaled prior to the analyses. Points represent plots. For model parameters, see Table 4

Regarding LMMs performed for plots representing the “*C*. *acutiformis* pathway,” all indices of functional diversity revealed significant decreases over time in both types of historical disturbance, with the most dramatic decreases reported for functional dispersion (with 44% of variance explained by random effects and 71% explained by both random and fixed effects) and functional divergence (in topsoil removal treatment; 14% of variance explained by random effects, and 18% explained by both random and fixed effects). The contribution of species with higher canopy heights, seed mass, and SLA increased significantly in the subsequent years of the experimental survey (approximately 14, 49, and 42% of the variance was explained by random effects, respectively; and 37%, 81%, and 65% was explained by both random and fixed effects, respectively). Only CWMs of seed mass and SLA revealed significant differences between treatments, where topsoil removal we identified as the treatment for which we observed the most pronounced increases in the proportion of plants with higher values of both functional traits over time (Figure 2; Table 2). Considering LMMs performed for plots representing the “*S*. *cinerea* pathway,” only functional divergence and functional dispersion decreased significantly over the five periods of the experimental survey (approximately 30 and 7% of the variance was explained by random effects, respectively; and 43% and 25% was explained by both random and fixed effects, respectively). While the magnitudes of temporal shifts in functional dispersion were similar in both treatments, decreases in functional divergence differed significantly between topsoil removal and mowing. In the same pathway, we found significant increases in the values of all CWMs of the functional traits analyzed, but only the CWM of SLA revealed significant differences between treatments (approximately 31% of the variance was explained by random effects; 63% was explained by both random and fixed effects), with a somewhat larger shift reported for topsoil removal (Figure 3; Table 3). Regarding the “*C*. *cespitosa* pathway” and functional diversity indices, we revealed no significant shifts in the subsequent years of the experiment and between treatments (except functional divergence, of which values decreased slightly over time). Regarding the LMMs performed for the CWMs of the functional traits, all of them revealed significant increases over time. While the magnitude of temporal shifts in the canopy height CWM (approximately 17% of the variance was explained by random effects and 50% was explained by both random and fixed effects) was similar in both experimental treatments, taking into account CWMs of seed mass and SLA, we observed different patterns. Considering the seed mass CWM (approximately 27% of the variance was explained by random effects and 47% was explained by both random and fixed effects), we reported the greatest increases in this parameter under conditions of historical topsoil removal, whereas the CWM of SLA (approximately 26% of the variance was explained by random effects and 44% was explained by both random and fixed effects) increased faster under conditions of historical mowing (Figure 4; Table 4).

**TABLE 2:** Parameters of linear mixed‐effect models showing changes in plant species functional diversity parameters (functional richness, divergence, and dispersion) and community‐weighted means (CWMs) of plant functional traits over time, and depending on historical disturbance type (fixed effects), with plot number adopted as a random factor, regarding the “*Carex acutiformis*” successional scenario: *SD*—standard deviation; *SE*—standard error; *t*—*t*‐statistics value; $R_{m}^{2}$—marginal coefficient of determination (the amount of variance explained by fixed effects only); $R_{c}^{2}$—conditional coefficient of determination (the amount of variance explained by both fixed and random effects). Significant results are in bold

Functional richness	Random effects	Variance	*SD*	Mixed model parameters	—
—	Plot number	0.379	0.616	$R_{m}^{2}$	.193
—	Residuals	0.491	0.700	$R_{c}^{2}$	.545
—	Fixed effects	Estimate	*SE*	*t*	Pr(>|*t*|)
—	(Intercept)	107.227	20.725	5.174	**<.001**
—	Historical disturbance	−0.324	0.089	−3.624	**<.001**
—	Year	−0.052	0.010	−5.081	**<.001**

Functional divergence	Random effects	Variance	*SD*	Mixed model parameters	—
—	Plot number	0.044	0.209	$R_{m}^{2}$	.144
—	Residuals	0.828	0.910	$R_{c}^{2}$	.187
—	Fixed effects	Estimate	*SE*	*t*	Pr(>|*t*|)
—	(Intercept)	97.053	21.523	4.509	**<.001**
—	Historical disturbance	−0.234	0.092	−2.521	.**012**
—	Year	−0.046	0.010	−4.364	**<.001**

Functional dispersion	Random effects	Variance	*SD*	Mixed model parameters	—
—	Plot number	0.292	0.540	$R_{m}^{2}$	.441
—	Residuals	0.300	0.548	$R_{c}^{2}$	.716
—	Fixed effects	Estimate	*SE*	*t*	Pr(>|*t*|)
—	(Intercept)	175.702	17.001	10.335	**<.001**
—	Historical disturbance	−0.379	0.073	−5.165	**<.001**
—	Year	−0.085	0.008	−10.131	**<.001**

CWM of canopy height	Random effects	Variance	*SD*	Mixed model parameters	—
—	Plot number	0.240	0.490	$R_{m}^{2}$	.141
—	Residuals	0.666	0.816	$R_{c}^{2}$	.368
—	Fixed effects	Estimate	*SE*	*t*	Pr(>|*t*|)
—	(Intercept)	−99.758	21.504	−4.639	**<.001**
—	Historical disturbance	−0.032	0.092	−0.346	.730
—	Year	0.054	0.010	5.060	**<.001**

CWM of seed mass	Random effects	Variance	*SD*	Mixed model parameters	**—**
—	Plot number	0.343	0.586	$R_{m}^{2}$	.487
—	Residuals	0.205	0.452	$R_{c}^{2}$	.808
—	Fixed effects	Estimate	*SE*	*t*	Pr(>|*t*|)
—	(Intercept)	−170.003	16.133	−10.537	**<.001**
—	Historical disturbance	0.470	0.069	6.756	**<.001**
—	Year	0.085	0.008	10.687	**<.001**

CWM of SLA	Random effects	Variance	*SD*	Mixed model parameters	—
—	Plot number	0.233	0.483	$R_{m}^{2}$	.423
—	Residuals	0.372	0.610	$R_{c}^{2}$	.645
—	Fixed effects	Estimate	*SE*	*t*	Pr(>|*t*|)
—	(Intercept)	−142.741	17.372	−8.216	**<.001**
—	Historical disturbance	0.505	0.075	6.733	**<.001**
—	Year	0.073	0.008	8.503	**<.001**

**TABLE 3:** Parameters of linear mixed‐effect models showing changes in plant species functional diversity parameters (functional richness, divergence, and dispersion) and community‐weighted means (CWMs) of plant functional traits over time and depending on historical disturbance type (fixed effects), with plot number adopted as a random factor, regarding the “*Salix cinerea*” successional scenario: *SD*—standard deviation; *SE*—standard error; *t*—*t*‐statistics value; $R_{m}^{2}$—marginal coefficient of determination (the amount of variance explained by fixed effects only); $R_{c}^{2}$—conditional coefficient of determination (the amount of variance explained by both fixed and random effects). Significant results are in bold

Functional richness	Random effects	Variance	*SD*	Mixed model parameters	—
—	Plot number	0.404	0.635	$R_{m}^{2}$	.009
—	Residuals	0.700	0.836	$R_{c}^{2}$	.372
—	Fixed effects	Estimate	*SE*	*t*	Pr(>|*t*|)
—	(Intercept)	−0.152	23.476	−0.007	.995
—	Historical disturbance	0.080	0.104	0.770	.443
—	Year	<0.001	0.011	0.078	.938

Functional divergence	Random effects	Variance	*SD*	Mixed model parameters	**—**
—	Plot number	0.142	0.377	$R_{m}^{2}$	.302
—	Residuals	0.599	0.774	$R_{c}^{2}$	.436
—	Fixed effects	Estimate	*SE*	*t*	Pr(>|*t*|)
—	(Intercept)	107.990	19.704	5.480	**<.001**
—	Historical disturbance	−0.502	0.087	−5.735	**<.001**
—	Year	−0.051	0.009	−5.274	**<.001**

Functional dispersion	Random effects	Variance	*SD*	Mixed model parameters	**—**
—	Plot number	0.188	0.434	$R_{m}^{2}$	.072
—	Residuals	0.783	0.885	$R_{c}^{2}$	.252
—	Fixed effects	Estimate	*SE*	*t*	Pr(>|*t*|)
—	(Intercept)	72.181	22.578	3.197	**<.01**
—	Historical disturbance	0.148	0.097	1.522	.130
—	Year	−0.034	0.011	−3.062	**<.01**

CWM of canopy height	Random effects	Variance	*SD*	Mixed model parameters	**—**
—	Plot number	0.527	0.726	$R_{m}^{2}$	.134
—	Residuals	0.439	0.663	$R_{c}^{2}$	.606
—	Fixed effects	Estimate	*SE*	*t*	Pr(>|*t*|)
—	(Intercept)	−95.685	21.729	−4.403	**<.001**
—	Historical disturbance	0.167	0.093	1.791	.075
—	Year	0.050	0.010	4.622	**<.001**

CWM of seed mass	Random effects	Variance	*SD*	Mixed model parameters	**—**
—	Plot number	0.106	0.326	$R_{m}^{2}$	.073
—	Residuals	0.850	0.922	$R_{c}^{2}$	.176
—	Fixed effects	Estimate	*SE*	*t*	Pr(>|*t*|)
—	(Intercept)	−74.027	22.576	−3.279	**<.01**
—	Historical disturbance	−0.074	0.097	−0.763	.446
—	Year	0.037	0.011	3.346	**<.01**

CWM of SLA	Random effects	Variance	*SD*	Mixed model parameters	**—**
—	Plot number	0.340	0.583	$R_{m}^{2}$	.311
—	Residuals	0.395	0.628	$R_{c}^{2}$	.630
—	Fixed effects	Estimate	*SE*	*t*	Pr(>|*t*|)
—	(Intercept)	−110.873	19.147	−5.791	**<.001**
—	Historical disturbance	0.515	0.082	6.246	**<.001**
—	Year	0.056	0.009	5.941	**<.001**

**TABLE 4:** Parameters of linear mixed‐effect models showing changes in plant species functional diversity parameters (functional richness, divergence, and dispersion) and community‐weighted means (CWMs) of plant functional traits over time and depending on historical disturbance type (fixed effects), with plot number adopted as a random factor, regarding the “*Carex cespitosa*” successional scenario: *SD*—standard deviation; *SE*—standard error; *t*—*t*‐statistics value; $R_{m}^{2}$—marginal coefficient of determination (the amount of variance explained by fixed effects only); $R_{c}^{2}$—conditional coefficient of determination (the amount of variance explained by both fixed and random effects). Significant results are in bold

Functional richness	Random effects	Variance	*SD*	Mixed model parameters	**—**
—	Plot number	0.292	0.540	$R_{m}^{2}$	.005
—	Residuals	0.800	0.894	$R_{c}^{2}$	.271
—	Fixed effects	Estimate	*SE*	*t*	Pr(>|*t*|)
—	(Intercept)	22.137	23.672	0.935	.351
—	Historical disturbance	−0.103	0.110	−0.937	.351
—	Year	−0.009	0.011	−0.827	.410

Functional divergence	Random effects	Variance	*SD*	Mixed model parameters	**—**
—	Plot number	0.132	0.363	$R_{m}^{2}$	.150
—	Residuals	0.768	0.876	$R_{c}^{2}$	.275
—	Fixed effects	Estimate	*SE*	*t*	Pr(>|*t*|)
—	(Intercept)	109.980	22.186	4.957	**<.001**
—	Historical disturbance	0.086	0.103	0.839	.403
—	Year	−0.053	0.011	−4.825	**<.001**

Functional dispersion	Random effects	Variance	*SD*	Mixed model parameters	**—**
—	Plot number	0.213	0.461	$R_{m}^{2}$	.050
—	Residuals	0.777	0.881	$R_{c}^{2}$	.254
—	Fixed effects	Estimate	*SE*	*t*	Pr(>|*t*|)
—	(Intercept)	−37.942	23.146	−1.639	.103
—	Historical disturbance	0.205	0.104	1.969	.051
—	Year	0.020	0.011	1.748	.082

CWM of canopy height	Random effects	Variance	*SD*	Mixed model parameters	**—**
—	Plot number	0.354	0.595	$R_{m}^{2}$	.173
—	Residuals	0.537	0.732	$R_{c}^{2}$	.502
—	Fixed effects	Estimate	*SE*	*t*	Pr(>|*t*|)
—	(Intercept)	−90.814	21.592	−4.206	**<.001**
—	Historical disturbance	−0.341	0.094	−3.598	**<.001**
—	Year	0.047	0.010	4.426	**<.001**

CWM of seed mass	Random effects	Variance	SD	Mixed model parameters	**—**
—	Plot number	0.175	0.418	$R_{m}^{2}$	.269
—	Residuals	0.471	0.686	$R_{c}^{2}$	.467
—	Fixed effects	Estimate	*SE*	*t*	Pr(>|*t*|)
—	(Intercept)	−122.101	18.495	−6.602	**<.001**
—	Historical disturbance	0.231	0.081	2.841	**<.01**
—	Year	0.061	0.009	6.688	**<.001**

CWM of SLA	Random effects	Variance	*SD*	Mixed model parameters	**—**
—	Plot number	0.086	0.293	$R_{m}^{2}$	.260
—	Residuals	0.259	0.509	$R_{c}^{2}$	.444
—	Fixed effects	Estimate	*SE*	*t*	Pr(>|*t*|)
—	(Intercept)	−85.130	13.506	−6.303	**<.001**
—	Historical disturbance	0.174	0.059	2.941	**<.01**
—	Year	0.043	0.006	6.401	**<.001**

## 4 DISCUSSION

We demonstrated that functional diversity of unmanaged wet meadow changed considerably over time. Regarding different pathways of succession, we revealed different magnitudes of compositional shifts. At the same time, the magnitudes of changes in functional diversity differed significantly between two types of historical disturbance and control. However, in case of some response variables we found relatively high amounts of variability explained by random factors linked with plot identity (e.g., functional richness, or CWM of canopy height in “*C*. *acutiformis* scenario”). Thus, interpretation of the patterns revealed should be made with a caution, bearing in mind that some of the results obtained may be biased by effects linked with pseudoreplications generated by our specific experimental design.

Results show that topsoil removal was a treatment in which temporal decreases in the values of functional diversity parameters (functional dispersion and functional divergence) were most pronounced and most rapid. Considering historical mowing, compositional shifts were gentler and of moderate magnitudes in comparison with topsoil removal. Similar tendencies, but with a positive direction of change, we found for CMWs of functional traits (seed mass and SLA), indicating the increasing importance of competition over time. This corresponds with results obtained by other authors (e.g., Niu et al., 2016; Schnoor et al., 2015), who found an increased proportion of plants producing heavier seeds and larger leaves after grazing or rotavation abandonment. In addition, under conditions of historical mowing, both taxonomic diversity and functional diversity were the highest. This can be expressed in weaker, in comparison with topsoil removal, rates of functional traits turnover, thus indicating less extended changes in the importance of habitat filtering, competition, and niche partitioning in shaping the community structure.

During the initial phase of the experiment, regardless of successional pathway, differences in the Bray–Curtis dissimilarity index between plots representing different treatments were the greatest. However, in the subsequent years of the vegetation resurvey, these differences decreased considerably, making the vegetation developing under topsoil removal more similar to mowing treatment and control. On the other hand, under topsoil removal during the initial phase of the experiment, we found the highest values of functional divergence (in the “*C*. *acutiformis*” and “*S*. *cinerea*” pathways). This suggests that directly after this strong disturbance had stopped, an area could be colonized by pioneer species, which may be expressed in large differences between extreme trait values carried by different species. On the one hand, this may indicate high role of random factors (linked with dispersal of propagules from surroundings) in shaping the colonization patterns in early successional stages, as well as hinting at important role for habitat filtering in shaping the structure of the vegetation (Tardella & Catorci, 2015; Waldén et al., 2017). This is in line with observations provided by other authors (e.g., Klimkowska et al., 2010; Resch et al., 2019), who found that after implementation of strong disturbances, more pioneer and/or specialized species can be promoted (Ludvíková et al., 2014; Schnoor et al., 2015). However, the group of pioneers can be rapidly replaced by plants with higher competitive abilities, characterized by higher SLA and seed mass CWMs (Niu et al., 2016; Waldén et al., 2017). In our case, values of these two functional traits revealed the most rapid increases in terms of topsoil removal. Similar tendencies were reported by Tardella and Catorci (2015), who found strong decreases in functional diversity after meadow abandonment, which was driven by an increase in the relative importance of competitive traits (e.g., canopy height and seed mass) in shaping the structure of plant communities.

After 20 years of the experimental survey, plant functional diversity of plots representing topsoil removal or mowing treatments became more similar to each other and to the control plots. These patterns may be evidence of functional homogenization (Cholewińska et al., 2020; Price et al., 2020; Reinecke et al., 2014; Sonnier et al., 2014), in our case expressed by simplification of plant functional diversity over time (O’Gorman et al., 2011). In this context, decreases in functional diversity could be linked with a decreasing number and abundance of specific combinations of functional traits carrying by different species (Mason et al., 2005). This may be indicated by increasing proportion of smaller number of more competitive plant species, with bigger aboveground biomass production and photosynthetic abilities, expressed, for instance, by temporal increases in the SLA CWM (Wright et al., 2004). Therefore, the observed signals of functional homogenization may be explained as a decreasing importance of habitat filtering and niche partitioning in species‐poor or species‐rich, but trait‐rich plots, and increasing the role of competition in shaping the community assembly (Graham & Fine, 2008; Webb et al., 2002). Thus, high number of pioneer and/or specialist species with unique combinations of functional traits (high trait divergence) may be replaced by low number of species with convergent traits. This loss of functional diversity may be expressed also by increasing functional redundancy (Sonnier et al., 2014),which in further perspective may exert key impacts on functioning of meadows, as well as alterations in ecosystem services provisioning (Cholewińska et al., 2020; O’Gorman et al., 2011; van der Plas et al., 2016).

When comparing different pathways of succession, we observed the largest and fastest decreases in functional dispersion and functional divergence in the “*C*. *acutiformis* pathway.” While the highest rate of decreases we reported for functional divergence under topsoil removal, in case of functional dispersion this decline was similar among both topsoil and mowing treatments. This provides evidence that plant species differing in the context of life strategies, of which existence in meadows may be determined by a large variety of specific microhabitats, could be eliminated by highly competitive species in subsequent years of the experiment. Therefore, the importance of niche partitioning in shaping the structure of vegetation could decrease, while the role of interspecific competition could increase over time (Rosenthal, 2010; Waldén et al., 2017). An explanation supporting this pattern may be a significant increase in the values of SLA and seed mass CWMs over time (Niu et al., 2016), suggesting rapid colonization by plant generalists from surrounding. One of example of such plant species may be *C*. *acutiformis*, which produces stolons with long internodes, which after three years of growth can reach approximately 200 meters of longevity, and produce over a thousand of ramets (Borkowska, 2004). This form of growth results in rapid, but diffuse occupation of large areas (Bernard, 1990). However, other authors reported the opposite tendency, demonstrating that seed mass was positively correlated with the implementation of mowing. This may be explained by trade‐offs connected with a larger allocation of resources used to increase seed mass than to investment in competitive traits under mowing, thereby diminishing competitive abilities (Otsus et al., 2014). Regarding functional richness, decreases in values of this parameter over time were less pronounced, but with similar rates of decreases among topsoil removal and mowing treatments, which may be additional evidence of the increased importance of the “competitive exclusion mechanism” in shaping the vegetation structure (Bernard‐Verdier et al., 2012). In this context, throughout the duration of the experiment, the degree of occupancy of the niche space by plants was relatively similar, which was probably linked with high species and functional trait turnover (Hedberg et al., 2014). Apart from *C*. *acutiformis*, the functional group of so‐called “competitive excluders” contained other tall graminoids or tall herbs with similarly high values of SLA and seed mass as *C*. *acutiformis*, as well as representing the guerrilla form of growth (Rosenthal, 2010). This strategy may enable fast colonization of highly disturbed habitat, dominance in biomass, and monopolization of the capacity for ecosystem services provisioning, which is in line with the “inhibition model” of succession (*sensu* Connel & Slatyer, 1977). This, in turn, may prevent the habitat from colonization by late‐successional species, at the same time driving large losses in the diversity of meadow target species (Waldén et al., 2017).

The “*C*. *cespitosa* pathway” we identified as the successional scenario with the least pronounced shifts in functional diversity, of which rates of changes were similar among topsoil removal, mowing, and control. This provides clear evidence that despite 20 years of secondary succession, niche partitioning could still be an important mechanism in shaping community assembly regarding the *C*. *cespitosa* pathway of succession (Carroll et al., 2011). The maintenance of a large diversity of different resource‐use strategies realized by a large number of “competitively inferior” species could be possible because of the potentially high variety of specific microhabitats created by *C*. *cespitosa*. This sedge represents the phalanx form of growth (Brzosko, 1999), and its large and old tussocks may constitute safe places for germination and growth of meadow specialists and pioneers. In this context, tussocks of *C*. *cespitosa* may play a role of nurse plants for the target meadow species. This may be achieved by provision of improved moisture and fertility conditions, as well as creation of sheltered microsites with presumably lower fluctuations of microclimatic conditions (Ballantyne & Pickering, 2015). Similar facilitating abilities were demonstrated for numerous shrub and tussocks species occurring, for instance, in extreme environments of alpine or arid zones (e.g., Navarro‐Cano et al., 2019). Under such microhabitat conditions, target meadow species can successively regenerate from this nonsoil seed bank deposited in the necromass formed by *C*. *cespitosa* (Borkowska, 2014). Similar tendencies were reported by Wang et al. (2019), who showed that old tussocks may be suitable microhabitats, where the probability of meadow species regeneration is high. On the other hand, the phalanx form of growth is characterized by ramets, which are located sufficiently close together. This, in turn, allows strong overlap of the resource depletion zones, but focussed on only one point in the patch (Harper, 1986). Therefore, remaining spaces between the tussocks of this sedge can constitute specific “light windows,” where the resource pool can still be high, and competition with guerrilla species from the surroundings may be of lower importance (Rosenthal, 2010). This is expressed by lower, in comparison to the “*C*. *acutiformis* pathway,” rates of temporal increases in the values of seed mass and SLA CWMs. At the same time, rates of these increases were relatively similar among treatments and control, revealing that regardless the historical disturbance type implementation, colonization of these patches by generalists of higher competitive abilities may be limited. On the other hand, it is likely that, in regard to the “*C*. *cespitosa* pathway,” extinction debt related to target meadow species could still not be paid, as specialist species can still exist in unmanaged meadows due to the high availability of efficient regeneration places (Otsus et al., 2014; Raatikainen et al., 2018).

*Salix cinerea* may play a similar nursing role for meadow target species as tussocks of *C*. *cespitosa*. This has been demonstrated for other shrub species, but occurring in more extreme environments of alpine or arid zones (e.g., Navarro‐Cano et al., 2019). However, the importance of *S*. *cinerea* for the creation of safe places for the regeneration and growth of meadow specialists may be lower than that of *C*. *cespitosa*. This may be linked with a significant increase in the proportion of species with higher SLA values and the production of heavier seeds, especially in terms of topsoil removal treatment. On the other hand, it may be connected with the encroachment of late‐successional species, that is, tall herbs or plants typical of forest understories (Kahmen & Poschlod, 2004). Another explanation of the patterns described above may be the fact that, in regard to the “*S*. *cinerea* pathway,” only the functional divergence revealed pronounced decreases over time, with the highest rate of decrease observed for topsoil removal. At the same time, functional dispersion decreased only slightly, reaching still relatively similar values in both treatments, comparing to control. Therefore, more specialized plants could decrease significantly in number and abundance (Kotowski et al., 2013), while the species for which the functional differences were smaller (Laliberté & Legendre, 2010) did not demonstrate strong negative responses over time. It is likely that this group of species contained plants typical of seminatural meadows, but that they were more like meadow generalists with presumably higher competitive abilities. This could prevent patches of vegetation developing under the canopies of *S*. *cinerea* from colonization by *C*. *acutiformis* and other inhibitors of succession, thereby buffering their negative impacts on functional diversity (Danet et al., 2018).

## 5 CONCLUSIONS

Our long‐term experimental survey contributed to a better understanding of the long‐term changes in the importance of ecological mechanisms in shaping the structure of unmanaged wet seminatural meadows in regard to different successional pathways and under different types of historical usage. We found that the implementation of both topsoil removal and mowing could promote the occurrence of both pioneer species and meadow specialists, thus maintaining the high importance of niche partitioning in shaping the community structure, especially in the “*C*. *cespitosa* pathway.” Therefore, the restoration of wet seminatural meadows in a scale of the studied region should focus more on the patches of vegetation that are dominated by tussock plants representing the phalanx form of growth or shrub (“*C*. *cespitosa* and *S*. *cinerea* pathways”), rather than sites where guerrilla species prevail (“C. *acutiformis* pathway”). Due to the potentially large role of nursing species, which may facilitate the maintenance and regeneration of meadow specialists, as well as buffer decreases in functional diversity (and thus prevent high niche partitioning), the potential restoration of meadows abandoned a long time ago may provide positive results, even regarding small spatial scales. Therefore, during the formulation of regional restoration plans of wet seminatural meadows, conserved in the Natura 2000 program, selecting these vegetation patches with implementation of a combination of mowing and topsoil removal, may substantially increase the probability of recovering most target species. At the same time, the application of this strategy may constitute a relatively fast and cost‐efficient method of meadow ecosystem restoration.

## CONFLICT OF INTEREST

The authors declare that they have no conflicts of interest.

## AUTHOR CONTRIBUTIONS

**Patryk Czortek:** Conceptualization (equal); Data curation (lead); Methodology (equal); Visualization (lead); Writing‐original draft (lead). **Lidia Borkowska:** Conceptualization (equal); Investigation (equal); Methodology (equal); Writing‐original draft (supporting). **Marlena Lembicz:** Conceptualization (equal); Investigation (equal); Methodology (equal); Writing‐original draft (supporting).

## Supporting information

Appendix S1

## Data Availability

The dataset analyzed in this study is accessible on the Dryad repository, https://doi.org/10.5061/dryad.d7wm37q13.
