# Supplementary material for: Long‐term shifts in the functional diversity of abandoned wet meadows: Impacts of historical disturbance and successional pathways
Source: Ecol Evol. 2021 Oct 12;11(21):15030–46. doi: 10.1002/ece3.8186 (PMC8571646; doi:10.1002/ece3.8186)

Supplementary Material for the paper:

Czortek P, Borkowska L, Lembicz M *Long term shifts in the functional diversity of abandoned wet meadows – impacts of historical disturbance and successional pathways*

Ecology and Evolution

**Appendix S1** Visualization of diagnostic plots performed for each vegetation parameter with respect to each successional pathway: ‘*Carex acutiformis*’(a), ‘*Salix cinerea*’(b) and ‘*Carex cespitosa*’ successional scenarios (c). Note that each diagnostic plot was performed based on one linear regression model without random factors, with vegetation parameters as response variables, and time and historical disturbance type as predictors. Each diagnostic plot visualizes the relationship between theoretical quantiles and standardized residuals.


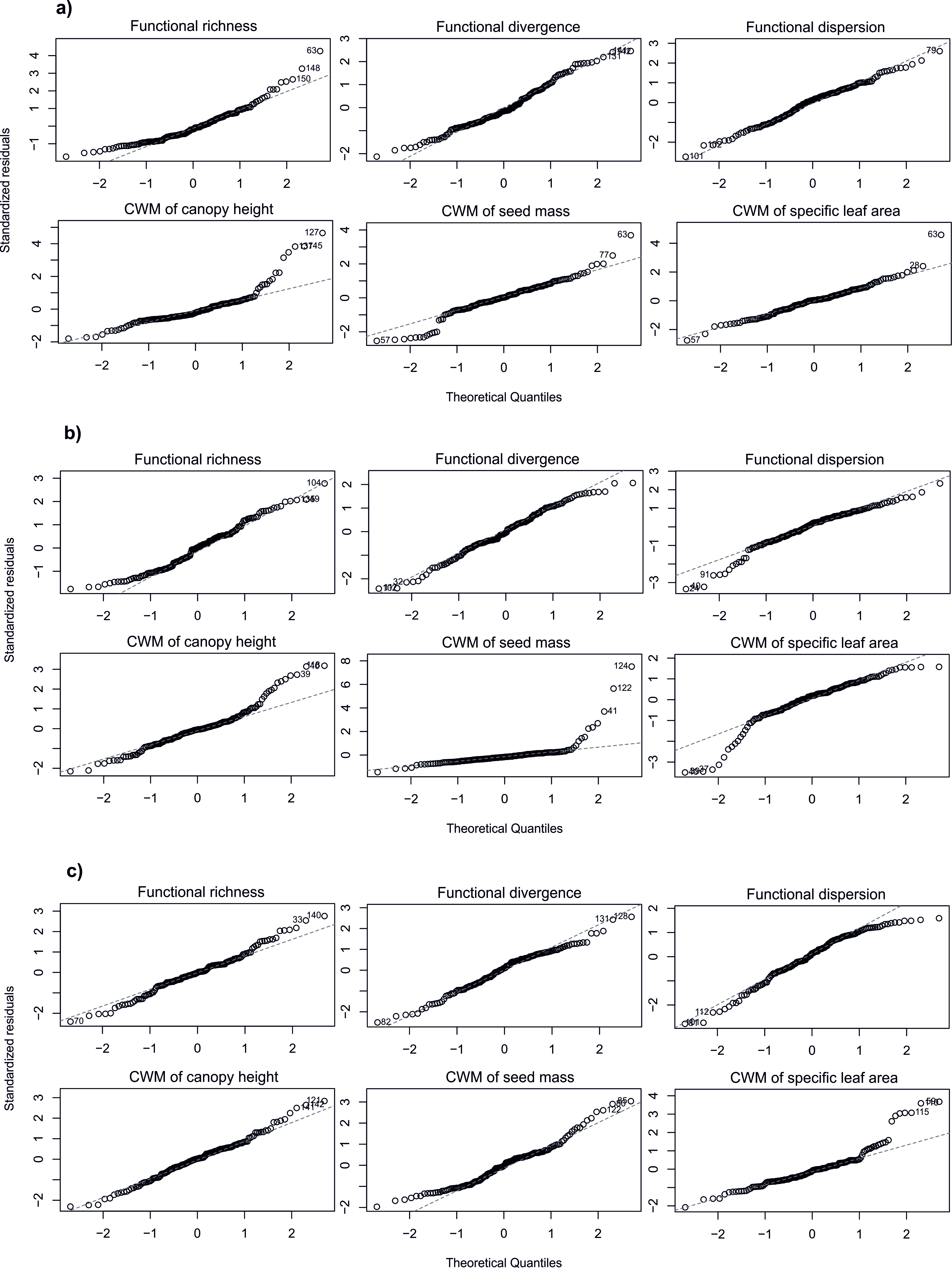

Supplement: Supplementary file 1 — Appendix S1 [file ECE3-11-15030-s001.docx]
